# Constraints to Implementing the Essential Health Package in Malawi

**DOI:** 10.1371/journal.pone.0020741

**Published:** 2011-06-14

**Authors:** Dirk H. Mueller, Douglas Lungu, Arnab Acharya, Natasha Palmer

**Affiliations:** 1 Department of Global Health and Development, London School of Hygiene and Tropical Medicine, London, United Kingdom; 2 Daeyang Luke Hospital, Lilongwe, Malawi; Kenya Medical Research Institute - Wellcome Trust Research Programme, Kenya

## Abstract

Increasingly seen as a useful tool of health policy, Essential or Minimal Health Packages direct resources to interventions that aim to address the local burden of disease and be cost-effective. Less attention has been paid to the delivery mechanisms for such interventions. This study aimed to assess the degree to which the Essential Health Package (EHP) in Malawi was available to its population and what health system constraints impeded its full implementation. The first phase of this study comprised a survey of all facilities in three districts including interviews with all managers and clinical staff. In the second and third phase, results were discussed with District Health Management Teams and national level stakeholders, respectively, including representatives of the Ministry of Health, Central Medical Stores, donors and NGOs. The EHP in Malawi is focussing on the local burden of disease; however, key constraints to its successful implementation included a widespread shortage of staff due to vacancies but also caused by frequent trainings and meetings (only 48% of expected man days of clinical staff were available; training and meetings represented 57% of all absences in health centres). Despite the training, the percentage of health workers aware of vital diagnostic and therapeutic approaches to EHP conditions was weak. Another major constraint was shortages of vital drugs at all levels of facilities (e.g. Cotrimoxazole was sufficiently available to treat the average number of patients in only 27% of health centres). Although a few health workers noted some improvement in infrastructure and working conditions, they still considered them to be widely inadequate. In Malawi, as in similar resource poor countries, greater attention needs to be given to the health system constraints to delivering health care. Removal of these constraints should receive priority over the considerable focus on the development and implementation of essential packages of interventions.

## Introduction

Policy recommendations to focus health care interventions on those that are considered most beneficial to the population originally stem from discussions around *selective* vs. *comprehensive* primary health care [1]. The first attempt on a global scale to prioritise interventions was reported in the World Development Report, 1993 [2], [3], backed up by the research carried out through the Disease Control Priorities Project (DCPP), and updated in 2006 [4], [5]. However, evidence as to how well such recommendations have been implemented or their impact within an existing health system is very limited until today [6], [7]. At the same time, there is growing interest in the role of the health system as a delivery mechanism for such interventions and how to overcome constraints that prevent its successful implementation [8].

The research detailed in this paper aimed to understand which health system factors constrained the delivery of the Essential Health Package (EHP) in Malawi. Malawi was chosen as the site for the research because it has a recently developed Essential Health Package (EHP), which, although not primarily guided by the DCPP, is largely congruent with the approach proposed by the DCPP.

The EHP in Malawi was conceptualised in 2002 to guide both planning and funding of health service delivery and to ensure an orientation towards the local burden of disease and mortality [9]. Moreover, a recent study confirmed the orientation of the EHP towards cost-effective health interventions responding to the local burden of disease [10]. Among the priority interventions targeted in the EHP were infectious diseases, including TB and HIV/AIDS (incl. opportunistic infections), adverse maternal and neonatal outcomes, nutritional deficiencies and common injuries; details of the EHP are described elsewhere [11], [12], [13].

A number of underlying constraints to the health system in Malawi were well known, such as insufficient stocks of essential drugs, inadequate infrastructure (including basic equipment, transport and communication), as well as a shortage of adequately trained and available health professionals [14], [15], [16], [17]. Recommendations alongside and as part of the EHP therefore called for strengthening of health centre and community level services, reducing costs of access (by providing EHP services free of charge), improving availability of drugs and equipment and strengthening performance of the district health system (through planning, budgeting, managing) [18]. Since the inception of the EHP in 2004 donor funds channelled through the Sector-wide Approach (SWAp) were intended largely at delivering the EHP interventions and rendering the health care system functional to deliver the package [19]. Concurrently, the health sector embarked on the process of decentralisation, which resulted in devolution of the drug budget to districts in 2006. However, until the end of 2010, the process of decentralisation had not been completed.

This research aimed to explore:

the extent to which selected EHP interventions were available and delivered at primary care level in facilities in three districts of Malawi;

the factors that constrained the delivery of these interventions at facility, level;

the role of district, zonal and national constraints.

Barriers to the full utilisation of the EHP exist both on the demand and the supply side. However, this research focussed on the health systems constraints which were on the supply side.

## Methods

Ethical and research clearance was applied for and granted by the Malawian National Health Sciences Research Committee and the Ethics Committee of the London School of Hygiene and Tropical Medicine.

**Figure 1:**
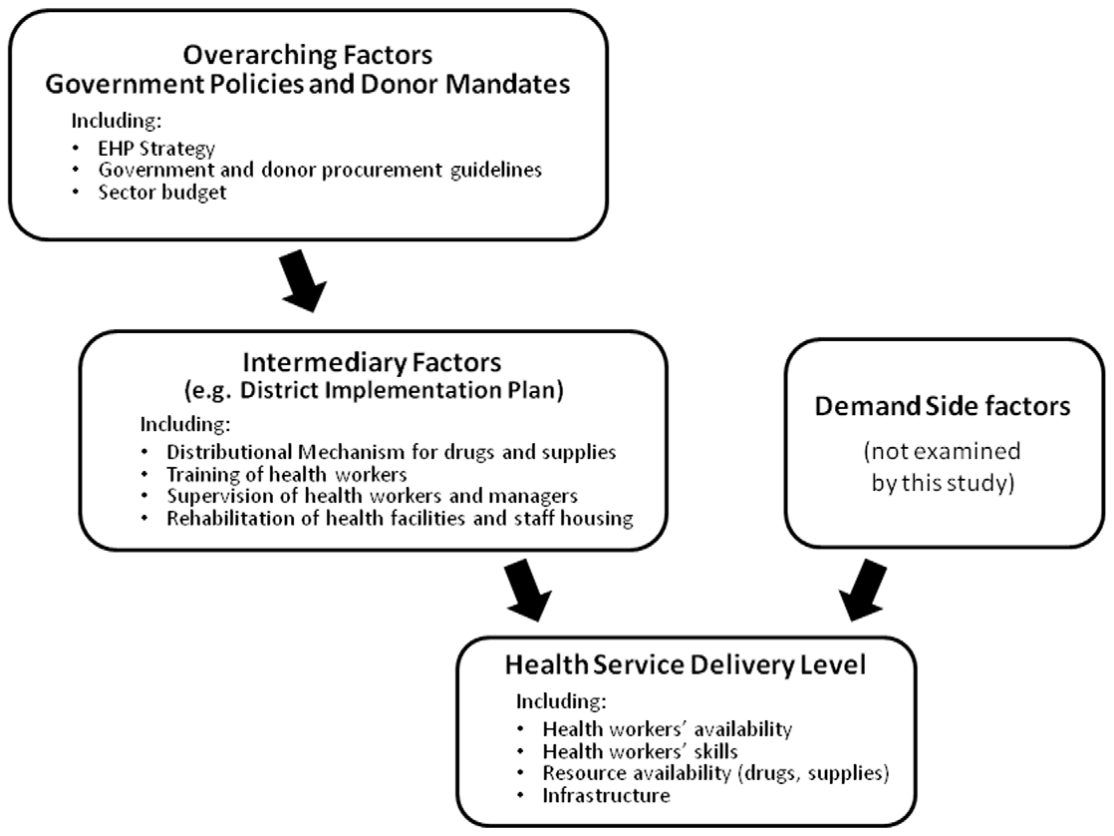
Conceptual framework – supplying health care in Malawi.

Figure 1 describes the conceptual framework underlying our study. It outlines the various inputs involved in health service delivery and where constraints are most likely to occur. Our investigation focuses on supply-side variables, which we have considered in three levels: Firstly and most immediately, health service delivery is determined by the availability and skills of health workers as well as the resources (in particular drugs) and infrastructure available to them. Secondly, on an intermediary level are institutional factors that impact on health workers' behaviour and support or constrain the availability of resources at facility level. These include drug distribution systems and frequency and modalities of training for staff. Thirdly, overarching factors at national level are made up of such factors as the EHP policies, budget, procurement mechanisms for medical supply and physical infrastructure. NGOs' and donor policies would also influence at this level. Both intermediary and overarching factors carry incentives and disincentives for the health worker and their management and therefore represent important influences to the quality of health care delivered.

The study design follows from the conceptual framework described above and is summarised in Table 1. The research was conducted during 2007 and 2008 in three phases.

**Table 1:** Study design.

Selection of Districts		
Nsanje	Nkhotokota	Rumphi
densely populated; difficult access to and within district (no tarmac, parts cut off during rainy season); southern most district – long distance from the capital; hot climate.	mid-level density; major tarmac road leading through it; most facilities within short distance to tarmac; long lake shore; easy access to the capital (2 hrs. drive); distinct burden of disease.	sparsely populated; district capital easily accessible but not other areas; moderate climate; Iong distance to capital but easily accessible (tarmac)
Description of Phases	Description	Distinct Feature
Phase I	Survey at the facility level, including interviews with health workers and the facility manager; All facilities visited (54 facilities with 136 health worker interviews)	37 rural health centres without maternity; 7 rural health centres with maternity service; 6 community rural hospitals; 3 district hospitals; 1 reproductive health care centre (NGO-operated)
Phase II	Solicitation of response to a presentation of findings in Phase I from the District Health Management Team (DHMT) and district health staff	Separate sessions for district health staff away from DHMT
Phase III	Key stakeholder interviews at the national level	Summary of findings in Phase I and II were sent before the interviews

In the first phase, a facility-level survey was performed at all facilities in three districts: Nsanje, Nkhotakota and Rumphi. These districts were chosen purposefully with the help of the Ministry of Health (MoH) to represent the country's largely rural ambience. The districts had varying accessibility and distance from the capital as well as different burden of diseases. This survey aimed to:

- assess the extent to which EHP services were available,
- explore factors limiting the delivery of these services.

The survey concentrated on four frequently occurring conditions covered by the EHP, selected to include acute conditions (1 and 2), long-term conditions (3) and maternal conditions requiring specific knowledge and equipment (4):

1. diagnosis and treatment of malaria (in children and adults),
2. acute respiratory tract infections (ARI),
3. opportunistic infections associated with AIDS (herpes zoster, Kaposi Sarcoma, oral thrush),
4. obstetric conditions/complications (post-partum haemorrhage, retained placenta, eclampsia; asked only to staff of facilities offering maternal services, n = 87).

The facility survey consisted of three parts:

1. A review of drug stocks. A checklist of drugs was developed based on the EHP drug list for the four tracer conditions. At the time of the facility survey (June–July 2007), a new policy on anti-malarial medication (towards artemesinin combination therapy, ACT) was in place, but had not been implemented in any of the districts under study. For malaria, the list was therefore amended to include the availability of Sulfadoxine/Pyrimethamine, SP, and Quinine, which were used at the time of the survey. A drug was considered to be sufficiently in stock if the amounts corresponded with the expected number of cases requiring this drug during the forthcoming three months according to the pharmacist/facility manager.
2. An interview with facility managers. All facility managers were interviewed on the services offered and numbers of consultations as well as, the facility's infrastructure and staffing levels (including absences and vacant posts).
3. A structured questionnaire with clinical staff at each facility. Staff were asked about aspects of their work experience at the facility using a structured questionnaire. All staff of health centres (except if absent for a more than 2 days) and up to 5 staff of each position in hospitals (doctor, clinical officer, medical assistant, registered and enrolled nurses) were interviewed. The questionnaire included an assessment of clinical knowledge related to the four tracer conditions. It also included statements about the working environment and feelings of the health workers which they were asked to either agree or disagree with using a Likert scale. Health Surveillance Assistants (community volunteers) were not interviewed as they are not considered clinical staff even if they perform some limited (presumptive) treatments in the community such as that of simple malaria [20].

Both manager and clinical staff interviews were conducted using a standardised, semi-structured questionnaire comprising predominantly closed questions. Both interviews ended with a small number of open ended questions (9 and 11, respectively) to elicit responses on qualitative aspects. The survey was conducted by Zonal Assistant Supervisors and Public Health graduates from the University of Malawi College of Medicine.

Three days were spent training and piloting of questionnaires before data collection started. All respondents were asked for prior consent to the interview and questionnaires were anonymised with respect to facilities and respondents. Answers on clinical knowledge were analysed with help of teaching staff from the University of Malawi Kamuzu College of Nursing. An answer was deemed sufficient if it met the minimum requirements to pass a comparable answer during exams of nursing students.

During Phase II, the results of this survey were discussed in each district with District Health Officers (DHO) and all available members of District Health Management Teams (DHMTs), and, separately, with senior district hospital staff.

During Phase III, individual meetings were held with national level stakeholders including key policy makers at ministerial level, the Central Medical Stores, representatives of NGOs and donor organisations. The selection of participants to these meetings followed recommendations by stakeholders in the Ministry of Health and tried to be as inclusive as possible. A written summary of the survey results was sent in advance to interview partners. Discussions during phases II and III were conducted in order to reflect on the results of the first phase and to solicit opinions why the identified problems and constraints existed.

## Results

### Phase I

An overview of the facilities visited and health workers interviewed during Phase I is given in Table 1. Of all health workers interviewed (n = 136), medical doctors and chief clinical officers represented 2% each, senior clinical officers 5%, clinical officers 4%, senior medical assistants 11%, medical assistants 22%, matrons 1%, senior registered nurses 7%, registered nurses 2%, senior enrolled nurses 24% and enrolled nurses 21%. The median number of years of professional experience for all positions was calculated at 13 years (interquartile range from 3 to 26 years).

According to clinical records, the 3 most common diagnoses among children under the age of 5 years as well as adults were malaria, ARI and gastroenteritis - consistent with the orientation of the EHP.

#### Awareness of the EHP

A first step in understanding the implementation of the EHP was to gain a sense of how well health service managers were aware of the policy. 44% of facility managers could explain the EHP in basic terms and stated that they ‘know about the EHP’. Among managers of health centres only, 33% knew of the existence of the EHP.

#### Drug availability

Most facilities reported problems with availability of drugs and key medical consumables. Table 2 shows the assessed availability of key drugs and consumables specified in the EHP for the 4 tracer conditions. For instance, 27% of the health facility managers considered Cotrimoxazole (to treat acute respiratory tract and other infections) to be of sufficient supply in their respective facility; 60% had insufficient stock, while in 13% of the facilities it was completely out of stock at the time of the survey. The situation for other oral antibiotics were even more critical with only 24% and 22% of health centres having sufficient amounts of Benzathine-Penicilline and Erythromycine in stock (with 13% and 20% entirely out of stock). The situation for SP 500 (at the time of the survey still the used regimen for simple malaria) was somewhat less severe with 42% of health centres disposing of sufficient stocks, and only 2% completely out of stock. Overall, hospitals were better equipped with drugs specified in the EHP. Moreover, in hospitals, there were more alternative drugs available, which were, according to pharmacists and clinicians, used when the original drug was out of stock.

**Table 2:** Percent of health centres (HC, n = 45) or hospitals (n = 9) which keep key drugs sufficient, insufficient or not at all in stock at the time of the survey (“sufficient” defined as the quantity deemed necessary by the pharmacist/facility manager to treat the average caseload that required this drug during the forthcoming 3 months; differences between HC and hospitals not statistically significant).

	Sufficient stock for 3 months to come		Insufficient stock for 3 months to come		Out of stock	
Drug	HC	Hospitals	HC	Hospitals	HC	Hospitals
Paracetamol	11%	22%	76%	78%	13%	0%
Aspirin	18%	33%	69%	67%	13%	0%
Diazepam	16%	33%	56%	67%	29%	0%
ORT	18%	22%	29%	56%	53%	22%
Nystatin oral drops	24%	33%	58%	56%	18%	11%
Chlorhexidine	36%	44%	27%	44%	38%	11%
Calamine lotion	44%	33%	38%	56%	18%	11%
Gentiana violet (GV)	51%	22%	31%	67%	18%	11%
Hydralazine	9%	0%	18%	56%	73%	44%
Cotrimoxazole	27%	44%	60%	56%	13%	0%
Metronidazole	18%	56%	62%	44%	20%	0%
Benz. Penicilline	24%	33%	62%	67%	13%	0%
Erythromycine caps.	22%	67%	58%	33%	20%	0%
SP 500	42%	33%	56%	67%	2%	0%
Quinine tablets	20%	11%	62%	89%	18%	0%
Quinine injectable	47%	67%	42%	33%	11%	0%
Lidocain	24%	11%	22%	33%	53%	56%
Syntometrine	42%	44%	38%	56%	20%	0%
Vitamin A	29%	11%	22%	22%	49%	67%
Magnesium sulphate	18%	56%	16%	11%	67%	33%

31% of facility managers indicated that they refer patients to a higher level of clinic due to unavailability of the required drugs.

#### Human Resource Availability

The percentage of envisaged health worker posts actually filled (as reported by the facility managers or District Health Offices) is given in Table 3. During the three months prior to the survey, 49% and 42% of expected clinical staff man days were available in health centres and hospitals, respectively. Unfilled positions account for the bulk of the shortfall in man days (82% and 87%). For those posts that were filled, trainings and meetings account for 57% of all absences in health centres and 52% in hospitals. Table 4 shows the number and percentage of workdays absent by reason. 52% of staff at hospitals and health centres had attended some training in the last three months. Those who had attended training had spent on average 16 (hospital) and 10 (health centre) workdays on training during the preceding 3 months. Of the staff that had undergone training, only 7% had requested that particular training.

**Table 3:** Percentage of expected positions filled by district and hc/hospitals (expected positions based on facility managers' and District Health Offices' information); HC n = 45, hospitals n = 9.

	Nkhotakota		Nsanje		Rumphi	
	HC	Hospitals	HC	Hospitals	HC	Hospitals
Physicians	50%	50%	n/a	33%	n/a	33%
Clinical Officers	67%	100%	n/a	52%	100%	37%
Medical Assistants	91%	100%	67%	73%	68%	65%
Regist. Nurses	56%	88%	n/a	33%	n/a	29%
Enr. Nurses	56%	78%	53%	56%	41%	60%

**Table 4:** Number of days absent per health worker (and percentage of total workdays) during 3 months by reason (n = total numbers of filled positions across all visited facilities).

	Physicians,n = 10	Clin. Officers, n = 52	Med. Assistants, n = 66	Reg. Nurses, n = 27	Enr. Nurses, n = 230
Leave	2.0 (3%)	3.8 (5%)	2.6 (4%)	2.6 (4%)	2.4 (3%)
Training	0.9 (1%)	3.1 (4%)	4.1 (6%)	6.6 (9%)	3.2 (5%)
Meetings	3.7 (5%)	3.0 (4%)	1.9 (3%)	2.3 (3%)	1.1 (2%)
Sick	0.2 (0.3%)	0.5 (1%)	0.9 (1%)	2.9 (4%)	0.9 (1%)
Relief and other reasons	0.5 (1%)	2.2 (3%)	2.2 (3%)	1.6 (2%)	1.6 (2%)
Total	7.3 (100%)	12.5 (100%)	11.7 (100%)	16.0 (100%)	9.3 (100%)

As a measure of staff workload, the median and mean numbers of patients per health worker per workday were calculated as 25.9 and 35.5, respectively, across all health centres (based on facility records and verified against district compilations). At hospitals, the respective median and mean numbers were 5.7 and 11.4.

#### Knowledge and capacity of staff

Knowledge and capacity of clinical staff was explored to shed light on the extent to which the personnel felt able and appeared able to treat the conditions in the EHP adequately. Among the health workers interviewed, 63% felt that they were adequately trained for their job (81% for hospital staff and 57% of health centre staff). 57% of health workers felt adequately supervised (46% in hospitals and 61% in health centres).

A selection of results of the knowledge assessment by hospital/health centre in all three districts is given in Table 5. An answer was considered “adequate” if it would obtain a pass mark for a similar question in a nursing exam. Where multiple answers were possible or required, adequacy was assumed even if only some of the elements were mentioned. While there is variation between districts, and between health centre and hospital staff, the results indicate a low level of knowledge in particular with respect to obstetric complications. Only 50% of health workers mentioned that they would check the respiratory rate to diagnose an ARI in children. For the treatment of a retained placenta, only 22% mentioned to empty the bladder and 60% raised the possibility of a manual removal. Some staff members also indicated that certain treatments could not be performed because of their lack of capacity and expertise.

**Table 5:** Percentage of health workers that mention selected key aspects for assessment and treatment of malaria, ARI, OI and obstetric complications (by hc/hospitals), n = total number of respondents, number of respondents to obstetric questions (with obstetric experience).

	Health Centres	Hospitals	Total
	n = 95,62	n = 36,25	n = 131,87
Malaria in children <5 assessment			
Anaemia	72%	83%	75%
Blood film	96%	97%	96%
Malaria in children <5 treatment			
SP	98%	100%	98%
Quinine	94%	100%	95%
Acute respiratory tract infections (ARI) assessment			
respiratory rate	47%	58%	50%
chest movements	69%	72%	70%
Acute respiratory tract infections (ARI) treatment			
Antibiotic Rx: Cotrimox., Xpen, Chloramphenicol	63%	81%	68%
Opportunistic Infections (OI) assessment: Zoster			
Dermatome distribution of symptoms	37%	69%	46%
Opportunistic Infections (OI) assessment: Kaposi Sarkoma			
Lesions on skin/in mouth	24%	42%	29%
Opportunistic Infections (OI) treatment: Zoster			
Analgesics	77%	89%	81%
Acyclovir	30%	69%	41%
Maternal Health (MH) assessment: Post-partum haemorrhage (PPH)			
Vital signs	53%	64%	56%
Contracted uterus?	58%	68%	61%
Maternal Health (MH) assessment: Retained placenta			
Bladder empty?	22%	20%	21%
Cervix open	12%	8%	11%
Maternal Health (MH) assessment: Eclampsia			
Vital signs/blood pressure	80%	80%	80%
Protein in urine?	53%	76%	60%
Maternal Health (MH) treatment: Post-partum haemorrhage (PPH)			
bi-manual compression	5%	8%	6%
Pitocin	23%	56%	33%
Maternal Health (MH) treatment: Retained placenta			
Empty bladder	25%	16%	22%
Manual removal	53%	76%	60%
Maternal Health (MH) treatment: Eclampsia			
MgSO4	22%	80%	39%
Hydralazine	13%	32%	19%

Selection of key aspects (not exhaustive).

#### Working and living conditions and staff satisfaction

Staff satisfaction was explored to gain insight on other dynamics in the experience of health workers in these facilities. Many health workers complained about their work and living conditions as well as their salary. Complaints concerned in particular the staffing and work load (some were on duty 24 hours for 7 days a week). Other difficulties included lack of basic equipment, means of communication, electricity, running water and in particular transport (for referrals). Although 39% of health workers had felt an improvement in working conditions, 66% of health workers would prefer to work at a different facility (health centres 71%, hospitals 54%). Overall, difficulties in working conditions (from infrastructure to supervision and referral) and perceived inadequate remuneration were mentioned to impact on the ability to cope with the demands placed on health workers especially in rural postings. Some staff members were outspoken about their fear of witnessing patients' deaths without being able to perform adequate treatment or to refer to higher levels of care in a timely manner.

### Phase II

In discussions at the district level, DHMTs recognised that training was a major reason for staff absences, some members stated:

“Sometimes we may have five trainings going on at the same time.” “There is no time for implementing the training because the trainings are continuous.”

To the question whether staff had been on training during the last two weeks, DHMTs responded that “*all of them have [been on training]*”. According to DHMTs, most training requests are either coming from central level or NGOs. While decentralisation was seen by some as enhanced empowerment (e.g. to decline training requests), one DHMT mentioned that sometimes the individual health worker or manager is informed prior to the DHO, which limits the discretion of the DHO to deny the training, and “*sometimes the Ministry will specify who they want to attend a particular training.*” All districts said that they refused training requests at times, but felt uneasy about doing so: “*Yes, but there are consequences.*” “*At times, we can send 5 instead of 6 [health workers].*” NGOs were particularly held responsible for uncoordinated training activities that are not based on training-need. DHMTs suggested that a central level regulation to limit the number of trainings would be helpful.

Various members of DHMTs pointed at the financial impact of trainings for the individual health worker. One DHMT mentioned that “*people scramble for trainings especially if there are financial incentives*”.

A further problem was that health workers who had attended training were not always successful in transmitting knowledge to their colleagues, in part due to jealousy from others who were not sent to the particular training and had to cover duties for those on training.

During discussions about the problems with drug availability, Central Medical Stores, Lilongwe (CMS) was largely held responsible for drug shortages. District Health Offices (DHO) are equipped with the discretion and resources to procure drugs on the private market following devolution in July 2006. However, *“districts' budgets are grossly insufficient to purchase all missing drugs from private pre-qualified suppliers”*. Some districts use other budget lines such as training, fuel or maintenance to subsidise drug purchases. Others try to collect unused drugs from their health centres in case of shortages at district hospital level, or, ask for drugs at neighbouring districts.

### Phase III

National level stakeholders were also not surprised at the high number of absent days due to training and meetings. Some donors recognised that they contributed to the frequency and volume of training activities. While central level stakeholders pointed out that all training activities should be included in the District Implementation Plan (DIP) drawn up by the DHO and DHMT, it was acknowledged that DIPs are often not followed. Instead, trainings are an important incentive for relevant managers to exercise control over their employees. There was little coordination and harmonisation of training activities, especially at central level.

One stakeholder summarised the view of various discussants: “Government is aware of the problem, but nobody has stood up to it so far”.

While most discussants agreed that fewer training, focussed on need, would be helpful, no stakeholder offered any comprehensive solution to the problem. It was mentioned that current government rules prevent re-structuring of training to reduce monetary incentives. Suggestions to improve the current ways of knowledge transfer include harmonisation of training activities between different actors; training should be provided on-site; pre-service training, but also increased supervision; rotation of staff; and improved emergency communication.

Concerning the availability of drugs, it was felt that that the facility survey may have coincided with a particularly difficult situation due to budgetary constraints at the end of the fiscal year. During the time when the survey was conducted there had been 6 emergency purchases by the Central Medical Stores (CMS). However it was also acknowledged that stock outs are not a rare occurrence. These appear to be due to a long list of challenges in the drug supply chain, including i) cumbersome and long World Bank procurement procedures, ii) insufficient qualified staff to perform quantification adequately, iii) a high turnover in staff, iv) poor warehousing and lack of space leading to drugs being pushed to the periphery too quickly and not based on need, v) poor inventory systems, and, vi) lack of capital funds to procure sufficient drugs in advance to cater for additional need and spoilage (buffer stock). The CMS appeared to take on the role of drug rationing as a response to these problems.

## Discussion

This study has detailed some of the barriers to implementing an essential or minimal health package in a setting such as Malawi. Although fewer than half of all interviewed health workers were able to explain the EHP, the correspondence was strong between the burden of disease as treated in the clinics, the staff's sense of what treatments are effective, and priorities identified in the EHP. This confirms a recent analysis [10]. Thus, there seemed little doubt over what the priority interventions in rural Malawi should be, whether this was referred to as part of the EHP or not. There were far greater barriers to ensure the delivery of these interventions, which was hampered by some severe shortages of key inputs.

This research has several limitations. The study focussed on four tracer conditions and not on the full EHP, and it concentrated on the supply side of delivering health care; the uptake of these services have been described elsewhere, e.g. [21]. Concerning the evaluation of vacancies, we relied on the expected number of staff by cadre as stated by facility managers and district health offices, as no conclusive planned staff allocation was available at national level at the time of the survey and even the established posts are not expected to necessarily reflect actual need [22]. However, the vacancy rates calculated in this study appeared to be reflect those described elsewhere [23], [24]. Despite these limitations, a number of findings were evident and confirmed during discussions with district and national level stakeholders.

The facility survey showed two key areas in which the health system was deficient for the delivery of the EHP: human resources and drug supply. Facilities fell short of even half of the expected staff allocations and staff, which was available, was often absent, especially on meetings and training. This is in line with findings of previous studies and reports [23], [24], [25]. In the case of training, per diems and allowances during training provide strong financial incentives for health workers, who struggle to make ends meet on their normal salary whereas allowances are often far beyond and in addition to the earned salary [16]. There is considerable competition for trainings among health workers, often leading to envy which hinders the sharing of the learnt material between other members of staff who were not offered the training. Because these allowances relate to trainings that are conducted off-site, external trainings are preferred by those attending the training and those financing the training wanting to ensure a high uptake. From the perspectives of donors, NGOs, and, partly, national stakeholders, trainings may be regarded as ‘quick-fix’ to introduce new programmes, standards or interventions. According to donors' own statements, donor organisations often have the ‘number of trained staff’ as a deliverable in their programme, where the aim of the training would not necessarily coincide with the perceived need by staff or DHOs. DHOs have a formal means to oppose this practice but would then stand in the way for their employees to receive the important financial incentives. Instead, management may use them in their own interest to motivate their employees. Some training activities, especially those organised by NGOs, are not integrated into national and district planning and may only be announced with short notice limiting rational planning by DHOs ahead of time. Lastly, the management has limited means to ultimately sanction health workers as they are highly dependent on its workforce, which is already in short supply.

According to our evaluation of knowledge, the trainings, however pervasive, did not appear to be very effective. Evaluations of the impact of these training are also not systematically pursued [26]. More adequate means to improve health workers knowledge and motivation, including closer and frequent supervision, on-site training and rotation of staff were mentioned by many discussants but have not been implemented sufficiently to date. Many rural health posts had not received supervision for months, and only few health workers identified the supervision they received as helpful in their clinical work. This points at the deficient human resource management (including the lack of continuous professional development) as identified in previous studies [16], [25], [26], [27], [28].

The availability of staff has to be considered in the light of workload and attendance rates. The number of patients per staff per day is within the range of similar settings in Sub-Saharan Africa or (in terms of hospital staff) even lower [29]. While one could speculate that the low attendance rates identified (of 1.4 visits per person per year) could be related to limited staff availability and perceived quality of service [21], our finding is higher than previously reported country-wide utilisation rates [25].

Shortages of essential drugs including vital antimalarials or antibiotics pervade all levels of care as had been documented previously in Malawi, even in the vicinity of the capital [25], [26], [30]. This excludes anti-retroviral drugs, which followed, up until this study, a different and independent mode of procurement and delivery [31], [32]). The reasons for inadequacies in drug procurement, storage and delivery were manifold. We documented deficiencies of finances, physical infrastructure (warehousing), staffing and drug quantification. Possibly a structural challenge to reform is that CMS is embedded in central government structures. This means that it is highly dependent on direct funding from the Ministry of Finance and has a lack of discretion over recruitment of staff (including their qualification) and inadequate means of responsibility to perform duties independent of central government. The devolution of purchasing power to districts is providing more discretion to districts but this measure will remain unsuccessful until CMS has the means to manage drugs adequately at national level, including quantification of need and keeping an adequate buffer stock. Many donors and others have therefore called for CMS to be changed into a (semi-) independent trust. Discussions regarding the institutional change are under way, but many complain about the long process and express doubt that CMS can ever become independent of political interference.

Although inadequacies of the health system were well known [14], [15], [16], [17], it appeared that insufficiencies of the health care delivery infrastructure were not sufficiently addressed before and while the EHP was put into place. Recent efforts have been made to re-design the package in terms of the number and type of services included. While this reform is arguably improving the planning and budgeting for the EHP with a more comprehensive costing as well as a further orientation at the burden of disease, our findings suggest that the degree of implementation of any package will be limited whilst the health system constraints remain as strong as they are in the case of Malawi.

### Conclusion

Our findings illustrate the challenge of implementing fundamental reform to health systems. We highlighted two areas that were particularly failing on the supply side of the EHP in Malawi – human resource management and drug availability. In both cases, the underlying causes of these failings are complex and involve not just a lack of resources but also intricate questions of governance, incentives and accountability. Whilst essential health packages present an important aspect of health system development that is relatively easily drawn up, solutions to the underlying problems of the supply side present a greater challenge.

Implementation of an essential or minimal health care package is not a panacea to the insufficiencies of a country's health care delivery system. An EHP is bound to the limitations set by its health care delivery infrastructure and system. In Malawi, as in other countries with a similar state of the health care system, the constraints to the health care delivery system must be prioritised over the design of such packages.
